# Molecular Characterization, Intra-Species Diversity and Abundance of Freshwater *Plesiomonas shigelloides* Isolates

**DOI:** 10.3390/microorganisms8071081

**Published:** 2020-07-20

**Authors:** Temitope Ekundayo, Anthony Okoh

**Affiliations:** 1 SAMRC Microbial Water Quality Monitoring Centre, University of Fort Hare, Alice 5700, Eastern Cape, South Africa; aokoh@ufh.ac.za; 2 Applied and Environmental Microbiology Research Group, Department of Biochemistry and Microbiology, University of Fort Hare, Alice 5700, Eastern Cape, South Africa; 3 Department of Biological Sciences, University of Medical Sciences, Ondo City PMB 536, Ondo State, Nigeria

**Keywords:** *Plesiomonas shigelloides*, DNA-fingerprinting, neighbor-joining, strains, molecular characterization, genetic diversity, intra-species barcoding

## Abstract

Molecular signatures of *Plesiomonas shigelloides* strain specific to pathogenic and nonpathogenic variants are not well established till present. There is a need for intra-species barcoding of *P. shigelloides* to aid infection control. This study aims at characterizing and assessing intra-species diversity and abundance of *P. shigelloides* isolated from three freshwaters in the Eastern Cape Province. The study used a Plesiomonas-specific PCR to characterize the isolates. Intra-species (dis)similarities were assessed using ERIC-PCR and (GTG)5-PCR techniques. The DNA fingerprints produced were electrophoresed, digitized, and documented via computer-assisted pattern analysis. The fingerprints were analyzed using neighbor-joining clustering (NJC) based on Euclidean similarity index. Results revealed 80%, 83.64%, and 80% of the water samples from Tyhume, Kat, and Kubusie rivers, respectively, positive for *P. shigelloides* isolation. The prevalence of *P. shigelloides* from sites ranged from 13.5% to 88.9%. NJC delineated 48 isolates to 8 clades (ERIC-fingerprints) and 34 isolates into 7 clades ((GTG)5-fingerprints). The relative abundance of unique strains ranged from 6.3% to 22.9% via the two methods. Both fingerprinting approaches have strain-differentiating potential for *P. shigelloides*, however ERIC-PCR possessed higher resolution (**D** = 37.46) advantage over (GTG)5-PCR (**D** = 29.64). In conclusion, the study achieved intra-species diversity and abundance of *P. shigelloides* from aquatic milieu and provide further opportunity for intra-species-specific barcoding.

## 1. Introduction

Molecular signatures of *Plesiomonas* strains specific to virulent and nonvirulent strains still defy understanding and remain a diagnostic challenge. With a clinical obligation to differentiate pathogenic strains from nonpathogenic variants, there is a need to understand intra-species/strain diagnostic features that would aid discrimination of a pathogenic strain of *P. shigelloides* from nonpathogenic counterparts. Generally, pathogenic strains of a bacterium species have unique genetic signature(s)/trait(s) that are not present in its nonpathogenic strains, thus, these signature(s)/trait(s) form the basis for differentiating virulent strains from avirulent strains. Presently, strain-typing of *P. shigelloides* irrespective of pathogenic potential relies on somatic (O) and flagella (H) antigens serotyping [1]. Improved development of *P. shigelloides* O/H antigens serotyping was advanced by Aldova [2] and Aldova and Schubert [3]. Unfortunately, a good number of *P. shigelloides* strains are not serotypeable [4]. Aside from the inability to serotype some strains, *P. shigelloides* also cross-react with *Shigella* species, resulting in a false-positive reaction [5]. Hence, serology is not a reliable way to type *P. shigelloides* strains. Also, *P. shigelloides* antisera is not commercially available [6]. The need for rapid turnaround diagnostics in both strain-typing and pathogenic strain delineation of *P. shigelloides* is eminent for infection control. A more recent method is still in the pipeline for detecting 12 serovars of *P. shigelloides* involving O-antigen-specific suspension array-based molecular typing [7] and still has the inability to surmount pathogenic and nonpathogenic discernment.

According to González-Rey et al. [8], genomic diversity of *P. shigelloides* isolates that shared similar serotype data from different sources has been reported using fingerprinting techniques. González-Rey et al. [8] disclosed that the pulsed-field gel electrophoresis (PFGE), random amplified polymorphic DNA-PCR (RAPD), repetitive extragenic palindromic-PCR (rep-PCR), and enterobacterial repetitive intergenic consensus-PCR (ERIC-PCR) delineated 24 strains involved in the study into 22, 21, 19, and 17 genotypes respectively, based on manual examination, thus suggesting the inadequacy in the use of serotyping in *P. shigelloides* strain discrimination. Shigematsu et al. [9] examined strain heterogeneity of *P. shigelloides* isolates using PFGE and DNA macrorestriction analysis in an epidemiological study of *P. shigelloides* travelers’ diarrhea in Kansai Airport, Japan. The study initially aimed at discriminating and detecting strains associated with disease severity and/or most prevalent in the specific locations. Although Shigematsu et al. [9] showed strain heterogeneity of the 39 *P. shigelloides* isolates and 3 reference strains via their PFGE fingerprints (each isolate had a unique profile and was highly variable), strain-specific signatures connected to disease severity were not reported or discoverable by the DNA profiles. Molecular fingerprinting only provides clues of strains’ diversities in the *P. shigelloides* isolates. In a similar manner, Gu and Levin [10] studied strain diversity among 6 fish, 10 freshwater’s samples, and 10 human clinical isolates of *P. shigelloides* using RAPD analysis. Genetic variability was notable among the majority of the isolates, unlike composite RAPD profiles. Some of the most outstanding results from the Gu and Levin [10] studies include notable similar RAPD profiles of isolates from the same source, similar profiles of the fish and the human clinical isolates, certain strains of the human clinical isolates with specific features, and genotypic similarity of freshwater and fish isolates, thus highlighting the possibility of cross-transmission among the various matrices. Another known method for assessing strain diversity such as 16S-23S ITS (Internal Transcribed Spacer) region in many microorganisms was further reported to be unable to detect heterogeneity amongst *P. shigelloides* strains [9]. Also, matrix-assisted laser-desorption/ionization time-of-flight mass spectrometry (MALDITOF MS) instrumental methods have been used for proteomics, structural and molecular characterization of *P. shigelloides’* lipopolysaccharide (LPS), and serotype (strain) delineation based on the O-PS side chain of LPS-variable chemical units as well as genetic variability in the *wb* cluster genes [11]. However, it has been shown that MALDITOF MS characterization of *P. shigelloides* lacks similarity between the same sero-strains [12].

Some authors believed the superiority of RAPD and PFGE as rapid, simple, and veritable sub-species typing techniques in strain-typing compared with restriction enzyme analysis, multilocus enzyme electrophoresis, and ribotyping [13], while many others have questioned their usefulness and reproducibility in the study of pathogens [14]. Notwithstanding that RAPD, rep-PCR, ERIC-PCR, RAPD, and PFGE have been used on *P. shigelloides* in different studies [8,9,13], many cogent questions about its strain diagnostic features remain unanswered. There also exist some unverified hypotheses about its pathogenicity potential. For instance, the proposition that the pathogenicity potential and/or the ability to cause gastroenteritis is ubiquitous to *P. shigelloides* isolates on the basis of the high variability of *Plesiomonas* chromosomal DNA patterns requires critical evaluation [9].

Generally, DNA-based techniques have been proposed by some authors to be characterized by high discriminatory power, high throughput, low cost, and considerable reliability of strain classification as well as typing of Gram-negative and Gram-positive bacteria [15]. The rep-PCR fingerprinting is a method in bacterial taxonomy that has been effectively applied in classifying lactobacilli [16], mycobacteria [17], staphylococci [18], vancomycin-resistant *Enterococcus faecium* strains [17,19], streptomycetes [20], and *E. faecium* clinical strains [21,22].

*P. shigelloides* is a single-species genus in the family Enterobacteriaceae [23]. It is well known to cause infections such as travelers’ diarrhea, gastroenteritis, to severe extraintestinal infections [9,24,25,26,27]. Also, some foodborne and waterborne outbreaks have been solely credited to *P. shigelloides* with sound microbiological and epidemiological validation [28,29]). Particularly, the incidence of *Plesiomonas* travelers’ diarrhea increased from 23.2% to 77.8% between 1987 and 1999 at Kansai Airport, Japan [9]. These and many more attest to the need to identify virulence signatures in *P. shigelloides* and the need for virulent strains’ diagnostic features. Strain-specific or virulent strain-specific diagnostic features would give room for discriminating its virulent strains from avirulent variants. Presently, no suitable phenotypic or molecular methods have been described for *P. shigelloides* virulent strain diagnosis.

From the foregoing, there is a need to understand and identify diagnostic traits of virulent and avirulent strains of *P. shigelloides*. However, the present study aims at characterizing and assessing intra-species heterogeneity and abundance of *P. shigelloides* isolated from aquatic environments in the Eastern Cape Province using molecular methods. This was aimed at enabling grouping of the strains to facilitate comparative studies of different groups to uncover the groups’ diagnostic traits. This approach will hopefully assist in extensive studies relevant for determining strain-specific molecular signatures of *P. shigelloides* that may provide insights for future endeavors in specific tagging of virulent (pathogenic) strains from avirulent (nonpathogenic) variants. For, we hypnotized that virulent strains of *P. shigelloides* have diagnostic trait(s) different from avirulent ones as it is common in other pathogens.

## 2. Materials and Methods

### 2.1. Cultural Isolation of P. shigelloides from River Water

*P. shigelloides* strains were isolated from 165 river water samples collected from freshwater resources at various points where human activities were prominent (see detailed sampling point descriptions in Table 1) across three popular rivers, viz. Tyhume, Kat, and Kubusie, in the Eastern Cape, South Africa. Sampling was done monthly and sequentially from the same sampling locations throughout February to December 2017. Water samples were collected aseptically in 1 L sterile glass bottle. Standard serial dilution of the water samples was carried out according to the standard protocol of APHA (American Public Health Association) [30]. Then, 100 mL aliquots of the respective dilutions were filtered through a 0.45µ millipore filter (Ø 47 mm) [30]. Each membrane filter, according to the dilution, was plated aseptically on a pre-labeled dried plate of inositol brilliant green bile agar (IBGBA) (HiMedia Laboratories, Mumbai-400086, India) using sterile forceps. After a period of 24 h incubation at 39 °C, pink colonies on the plates were counted and recorded as presumptive *P. shigelloides* isolates. Some randomly selected pink colonies were further streaked on a fresh IBGBA to purify them, subsequently grown on nutrient agar, and assayed for oxidase enzyme production using oxidase strips [31]. Oxidase-positive isolates were stored on glycerol stocks (−80 °C) for further studies.

### 2.2. DNA Extraction and Molecular Characterization of P. shigelloides

The purified oxidase-positive isolates were re-streaked onto fresh nutrient agar plates and cultured overnight at 37 °C. Total DNA of the 24 h culture was extracted by direct boiling procedure [32]. Two to three single colonies of the 24 h culture were picked and reconstituted in 200 µL sterile distilled water by vortex using a vortex mixer (DiGiSystem Laboratory instruments INC, Taiwan, Republic of China (**ROC**). The reconstituted cells were washed repeatedly with three changes of sterile DH_2_O at 15,000 rpm/2 min. The final cell pellet was re-suspended in nuclease-free water and boiled (100 °C/10 min) using Dri-Block^®^DB-3D (Bibby Scientific LTD, Staffordshire, UK). The boiled cells suspension was then centrifuged at 15,000 rpm/10 min in a micro-centrifuge (HEMLE Labrtechnik GmbH, Germany) to separate cell debris. The supernatant (DNA) was collected in a sterile Eppendorf tube and stored in a freezer (−20 °C) until further use.

The presumptive isolates were characterized using *Plesiomonas*-specific 23S rRNA polymerase chain reaction [4]. The primer used was PS23FW3 5′-CTCCGAATACCGTAGAGTGCTATCC-3′ and PS23RV3 5′-CTCCCCTAGCCCAATAAC ACCTAAA-3′, with the expected amplicon product of 284 bp (Gu and Levin, 2006) [10]. The PCR reaction consisted in 10× reaction buffer (2.5 µL), dNTPs (1 µL of 2.5 mM), MgCl_2_ (1.25 µL of 50 mM), primers (0.6 µL each, 10 µM), Taq polymerase (0.1 µL of 5 u/µL), 2.5 µL deoxyribonucleic acid of each isolate, and 16.45 µL sterilized nuclease-free water in a 25 µL reaction volume. The negative control was devoid of any DNA template. The thermal scheme for the reaction included initiation (1 cycle, 95 °C, 5 min), 35 cycles of denaturation (94 °C, 1 min), annealing (68 °C, 1 min), and extension (72 °C, 1 min), and final extension (72 °C, 10 min). Five microliters (5 µL) of the amplicons from each tube was electrophoresed in a 1% w/v agarose gel (Laboratorois Conda, Madrid, Spain). The gel had 2 µL ethidium bromide (0.2 µg/mL) incorporated. All electrophoresis was performed in a TBE (Tris-borate-EDTA) buffer (pH 8.0) (0.089 M Tris, 0.089 M boric acid, and 0.002 M EDTA (ethylenediaminetetraacetic acid) at 100 V/45 min (2.22 V/min). A 100 bp DNA standard served as gene ruler for the gel. At the completion of electrophoresis, bands were visualized using UV trans-illumination and photographed.

### 2.3. Fingerprinting, Fingerprint Treatment, and Computer-Assisted Analysis

Molecular diversities of selected, confirmed *P. shigelloides* isolates were determined by rep-PCR [33]. Two sets of primers including ERIC1 5′-ATGTAAGCTCCTGGGGATTCAC-3′, ERIC2 5′-AAGTAAGTGACTGGGGTGAGCG-3′, and (GTG)_5_ 5′-GTGGTGGTGGTGGTG-3′ were used. The reactions for the two sets of primers were carried out in 20 µL total reaction volumes, made up of 1 µL of ERIC primer or 2 µL of (GTG)_5_ primer, 1.5 µL of MgCl_2_, 10 µL of the master mix, 2 µL of isolate’s DNA template, and 3.5 µL of sterilized nuclease-free water. While the thermal programs for ERIC-PCR consisted of initiation (94 °C/6 min), 35 cycles of denaturation (94 °C/0.5 min), annealing (48 °C/1 min), extension (72 °C/5 min), and final extension (72 °C/7 min), the thermal program for (GTG)_5_-PCR likewise comprised initiation step (94 °C/4 min), 40 cycles of denaturation (94 °C/1 min), annealing (40 °C/2 min), and extension (72 °C/2 min); and final extension (72 °C/10 min). Five/ten microliters (5/10 µL) of the ERIC-PCR and (GTG)_5_-PCR products was electrophoresed using TakaRa Mupid-ONE (Takara Bio Inc, Shiga, Japan). A 1.5% agarose gel (Laboratorois Conda, Madrid, Spain) with 2 µL ethidium bromide (1 µg/mL; Sigma-Aldrich, USA) incorporated in a TBE buffer (pH 8.0) comprising 0.089 M boric acid, 0.089 M Tris, and 0.002 M EDTA) was used. Equivalent volume of the PCBIO ladder (PCR Biosystems Ltd., London, UK) made up of a mixture of marker sizes ranging from 100 bp to 10 kb was filled into terminal of the gel. The gel was visualized for DNA fingerprints and documented using a UV transilluminator.

All fingerprint images were digitized for a computer-assisted pattern analysis using GelJ version 2.0 software [34]. The DNA fingerprints occurrence matrices and molecular weight of ERIC2-PCR and (GTG)_5_-PCR bands were generated from their gel images using the unweighted pair group arithmetic mean algorithm at 1.0% tolerance level for quality control.

### 2.4. Assessment of Intra-Species/Strain Diversity of P. shigelloides Isolates

The band occurrence matrices of (GTG)_5_-PCR and ERIC-PCR fingerprints generated by the GelJ 2.0 software were imported into PAleontological Statistics Version 3.23 (PAST3.23) [35] for diversity studies. The absence/presence of a fingerprint across the isolates formed the basis for strain homogeneity or heterogeneity (associations) assessment. Firstly, dendrograms of the two matrices were created by neighbor-joining (NJ) [36] using a Euclidean similarity index (Equation (1)).

(1) $$d_{jk}=\sqrt{\underset{i}{\sum }{\left(x_{ji}-x_{ki}\right)}^{2}}$$

Here, NJ defines the distance between any strain pair *i* and *j* as *d_ij_* that minimizes *Q* criterion (7.2).

(2) $$Q_{i,j}=\left(r-2\right)d_{i,j}-\overset{r}{\underset{k=1}{\sum }}d_{i,k}-\overset{r}{\underset{k=1}{\sum }}d_{j,k}$$

where r is the current number of bands (representing strains) and the sums run on the band (strain) set. Secondly, the abundance of strains that form a clade was computed. Then, the study compared the resolution power of each fingerprinting technique using the Shannon’s index (H) and Simpson’s index based on the number of clades from the neighbor-joining clustering (NJC).

## 3. Results

**Figure 1:**
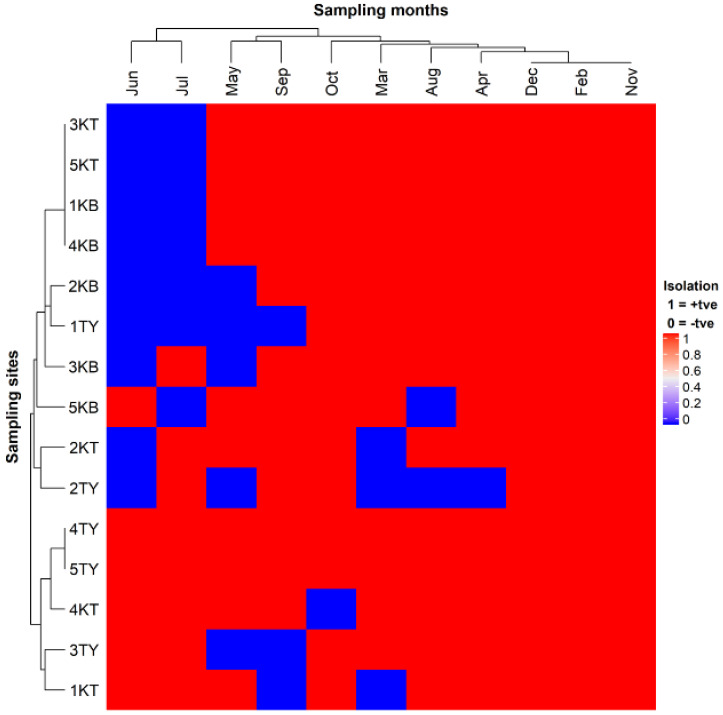
Heatmap of cultural detection of *P. shigelloides* across months in water samples collected from selected rivers in the Eastern Province. Red color represents positive isolation of *P. shigelloides,* while blue color represents no isolation. 1KB, Stutt. Game Reserve*; 1KT, Seymour; 1TY, Hogsberg; 2KB, StuttTW1*; 2KT, Katberg; 2TY, Hillfoot*; 3KB, StuttVBridge1*; 3KT, Balfour; 3TY, Kayalethu; 4KB, StuttEbBridge*; 4KT, Blinkwater; 4TY, Binfield; 5KB, StuttFbridge*; 5KT, Fort Beaufort; 5TY, Melani. * Arbitrary name.

Fifty-five samples were processed for each of the three sampled rivers during the eleven months (February–December 2017). From Tyhume, Kat, and Kubusie rivers, 80% (44/55), 83.64% (46/55), and 80% (44/55) of samples were positive for *P. shigelloides,* respectively. Overall, 134 (81.21%) out of 165 samples examined from the rivers yielded *P. shigelloides* (Figure 1).

**Figure 2:**
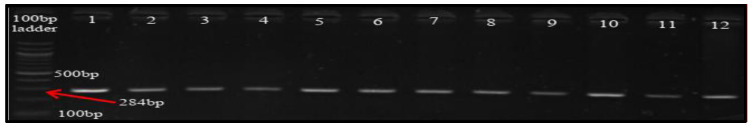
A representative *P. shigelloides*-specific 23S rDNA gel image. Line 1 = Positive control (*P. shigelloides* DSM 8224); lines 2 to 12 = positive isolates.

Seven hundred and forty-eight randomly selected presumptive colonies of *P. shigelloides* were oxidase-positive (Table 2). Out of these, two hundred and eleven isolates (n = 211, 28.21%) were identified as *P. shigelloides* by *P. shigelloides*-specific 23S rRNA polymerase chain reaction. These 211 *P. shigelloides* isolates yielded a band of 284 bp that confirmed their identity as *P. shigelloides* [4]. The sites 2TY and 5KT contributed 1.42% (lowest) and 18.01% (highest) of the total *P. shigelloides* confirmed from the rivers, respectively. A representative *P. shigelloides*-specific 23S rRNA gel image is shown in Figure 2.

**Figure 3:**
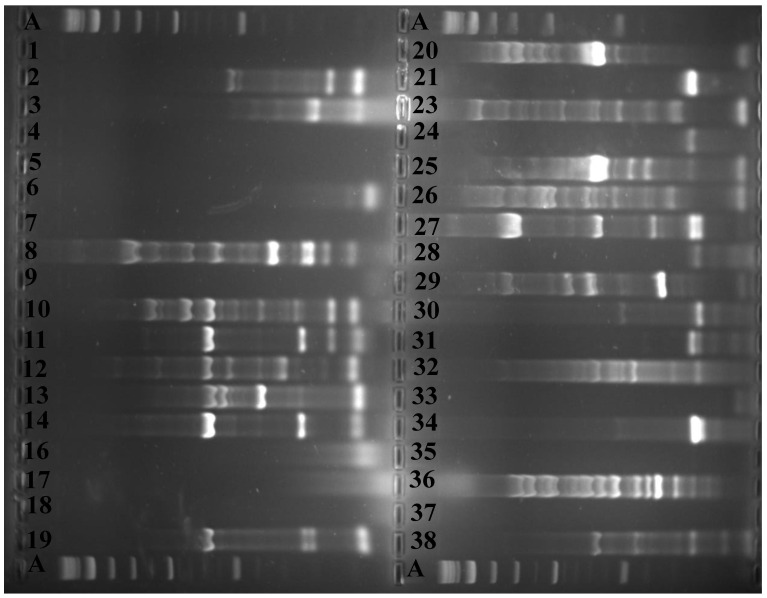
A representative ERIC-PCR fingerprint gel image. A = 1kb molecular ladder, lines 1 to 38 = *P. shigelloides* isolates.

**Figure 4:**
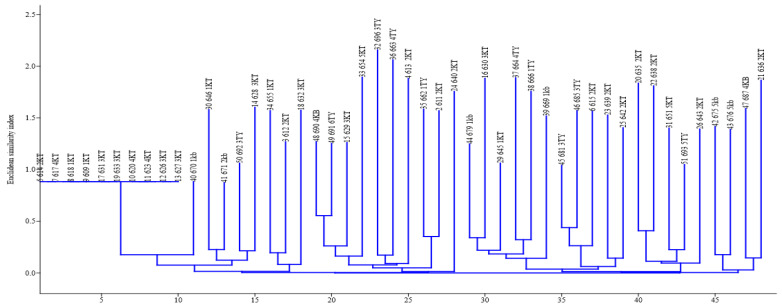
Neighbor-joining dendrogram clusters of ERIC-fingerprints of *P. shigelloides* strains. Isolates were encoded, for example, 32 696 3TY representing strain number (32), assigned number for PCR confirmed (696), and origin of the isolate (3TY).

A representative ERIC-PCR fingerprint image is presented in Figure 3. The ERIC-PCR banding patterns of isolates ranged from 0 to 10 bands. Molecular weight of bands also varied from 380 to 5665 bp (Figure 3). Certain isolates did not produce any band and appeared not typeable by the ERIC-PCR. The ERIC-PCR fingerprints dendrogram constructed by NJ using a Euclidean similarity index is shown in Figure 4. All the isolates clustered together. However, eight clades of strains were observed. The clades were as follows, numbering from origin (0) along the horizontal axis: clade 1: 614 2KT, 617 4KT, 618 1KT, 609 1KT, 631 3KT, 633 3KT, 620 4KT, 626 3KT, 627 3KT, and 670 1Kb; clade 2: 646 1KT, 671 2Kb, 692 3TY, and 628 3KT; clade 3: 655 1KT, 612 2KT, and 632 3KT; clade 4: 690 4KB, 691 6TY, 629 3KT, 654 5KT, 696 3TY, 663 4TY, 613 2KT, 662 1TY, 611 2KT, and 640 2KT; clade 5: 679 1KB, 630 3KT, 645 1KT, 664 4TY, 666 1TY, and 667 1Kb; clade 6: 681 3TY, 685 3TY, 615 2KT, 639 2KT, and 642 2KT; clade 7: 635 2KT, 638 2KT, 651 5KT, 693 5TY, and 643 2KT; and clade 8: 675 5Kb, 6765Kb, 687 4KB, and 636 2Kb. The relative abundance (%) of the isolates that made up each clade was 22.9% (11/48), 8.3% (4/48), 6.3% (3/48), 22.8% (10/48), 12.5% (6/48), 10.4% (5/48), 10.4% (5/48), and 8.3% (4/48) for clade1, clade 2, clade 3, clade 4, clade 5, clade 6, clade 7, and clade 8, respectively. ERIC-PCR strain typing resolution power was estimated as 17.39 (H) and 37.46 (D).

**Figure 5:**
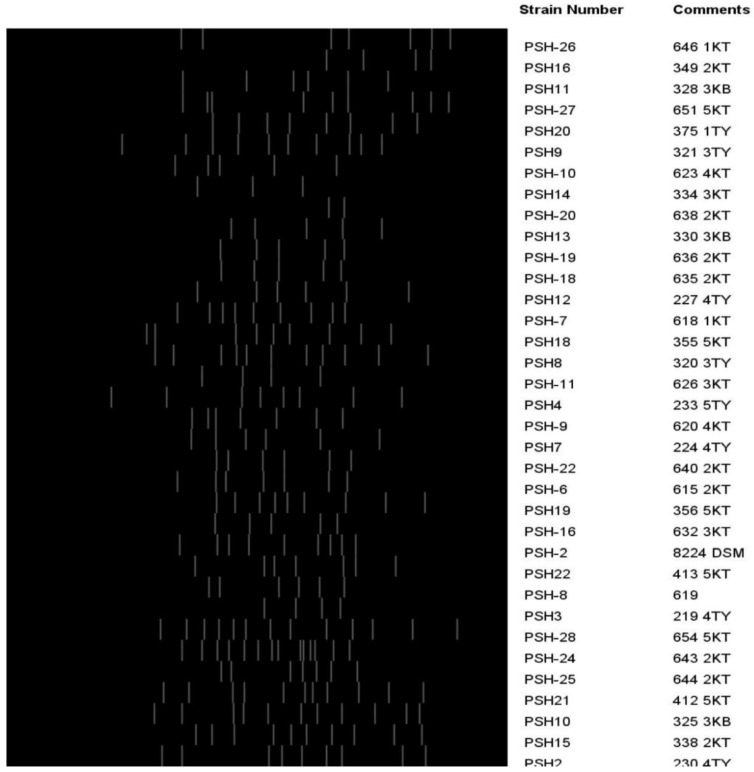
A (GTG)5-PCR fingerprint digitized image. Isolates were encoded, for example, PSH-26 646 1KT, representing strain number (PSH-26), assigned number for PCR confirmed (646), and origin of the isolate (1KT).

**Figure 6:**
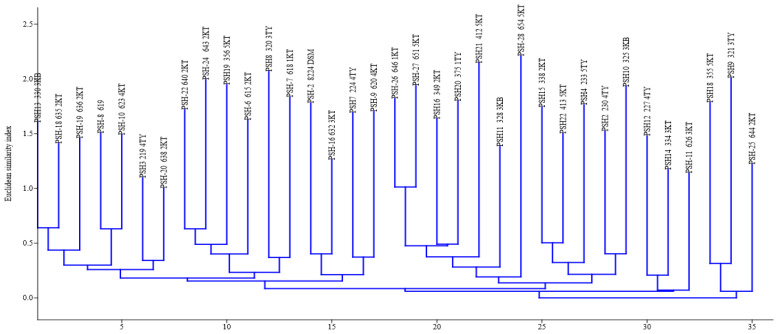
A neighbor-joining dendrogram cluster of (GTG)5-PCR fingerprints of *P. shigelloides* strains. Isolates were encoded, for example, PSH-15 338 2KT, representing strain number (PSH-15), assigned number for PCR confirmed (338), and origin of the isolate (2KT).

A typical digitized (GTG)5-PCR fingerprint image is presented in Figure 5. (GTG)5 fingerprints produced band patterns that ranged from 4 to 14. The (GTG)5 fingerprint bands ranged in size from 147.76 to 5304.98 bp. All the isolates produced (GTG)5 fingerprint bands and clustered together. The NJ clustering of (GTG)5 fingerprint resulted in seven clades. The composition of each clade, numbering from origin (0) along the horizontal axis to 35 scale, was as listed (Figure 6): clade 1: 330 2KB, 635 2KT, 636 2KT, PSH-8 619, 623 4KT, 219 4TY, and 638 2KT; clade 2: 640 2KT, 643 2KT, 356 5KT, 615 2KT, 320 3TY, and 618 1KT; clade 3: PSH-2 8224 DSM, 632 3KT, 224 4TY, and 620 4KT; clade 4: 646 1KT, 651 5KT, 349 2KT, 375 1TY, 412 5KT, 328 3KB, and 654 5KT; clade 5: 338 2KT, 413 5KT, 233 5KT, 233 5TY, 230 4TY, and 325 3KB; clade 6: 227 4TY, 334 3KT, and 626 3KT, and clade 7: 355 5KT, 321 3TY and 355 5KT, 321 3TY, and 644 2KT. The relative abundance (%) of strains making up each clade was 20.6% (7/34), 17.7% (6/34), 8.8% (3/34), 20.6% (7/34), 414.7% (7/34), 8.8% (3/34), and 8.8% (3/34) for clade 1, clade 2, clade 3, clade 4, clade 5, clade 6, and clade 7, respectively. (GTG)5-PCR fingerprint resolution power was calculated as 13.76 (H) and 29.64 (D).

## 4. Discussion

High detection (positive) rates of *P. shigelloides* were observed in the sampled waters in this present study. The *P. shigelloides* cultural prevalence rates of 80% (44/55), 83.64% (46/55), and 80% (44/55) observed in river water from Tyhume, Kat, and Kubusie, respectively, were higher than those in earlier reports from river water in literature.

The observed high recovery rate compared with previous studies in part could be attributed to a combined use of filtration technique and Inositol Brilliant Green Bile Agar for the isolation of *P. shigelloides* in this study. Most previous studies used some other media and culture techniques [37,38] that allowed *P. shigelloides* to compete with a host of other bacteria, which, in most cases, have higher growth advantages in the media compared with *P. shigelloides*. Some rates of positive detection of *P. shigelloides* from river, well, or pond water in other works include 40.7% [37], 16.67% (4/24) in Rio de Janeiro City in Brazil [38], 0.6% (well) and 7.4% (pond) in Zaria, Nigeria [39], 12.8% in Japan [40], and 13.3% in Dhaka, Bangladesh [41].

The overall *P. shigelloides* yield of 81.21% (134/165) from river water observed in this study could have arisen from probably organic and inorganic contaminants in the rivers that support the growth of *P. shigelloides*. For instance, herbicides, fertilizers, and pesticide input into the rivers occurs frequently at 3TY, 5TY, 2KT, 4KT, 3KB, and 5KB. More so, anthropogenic resuspension of the riverbeds, as frequently observed in the area during animal watering in the sites, might increase the levels of *P. shigelloides* in the overlying water because sediment resuspensions release *P. shigelloides* trapped in riverbeds, biofilms, and other matrices into the water column. *P. shigelloides* has been isolated from pond sediment, hydrophytes, and phytoplankton [41]. Some authors have reported isolation rates of *P. shigelloides* from freshwater sediment, hydrophytes, and phytoplankton as 29.2%, 20.8%, and 18.3%, respectively [41]. Islam et al. [41] noted that matrices associated with pond such as soil, sediment, phytoplankton and hydrophytes had 62.5%, 41.7%, and 33.3% *P. shigelloides* positive isolation rate in their study.

Generally, the choice of IBGA and membrane filtration in isolation encouraged the recovery performance of *P. shigelloides* in this study. The use of *Plesiomonas* isolation agar and membrane filtration for cultivation of *P. shigelloides* from the Nilufer Stream in Bursa, Turkey found an 83% (30/36) positive rate [42]. Most media for isolation of coliform and members of the Enterobacteriaceae allow the growth of *P. shigelloides* with varying recovery efficiencies and specificities. These media include deoxycholate-hydrogensulphide lactose agar [38], Salmonella-Shigella agar [34,43,44], MacConkey [43,45,46], Hektoen enteric agar [45], modified Salmonella-Shigella agar [40,44], deoxycholate citrate agar [46], taurocholate tellurite gelatin agar [41], xylose lysine deoxycholate agar [39,45], Endo agar [47], Plesiomonas differential agar [37,41], and peptone inositol bile and broth tetrathionate broth without iodine [37].

Another possible explanation for the high recovery rate of *P. shigelloides* from the sampled rivers without enrichment in this study could be due to favorable water temperature. The average temperature of the rivers ranged from 4.7–25.8 °C during the sampling period. The relative high water temperatures, known to be favorable for *P. shigelloides* multiplication, may have resulted in the higher rate of detection. However, isolation of *P. shigelloides* from freshwater water samples in the temperate and colder regions of the world, such as Czech Republic [48], Hungary [37]; the Netherlands [49], Slovakia [50], subpolar region of Sweden [47,50], and Serbia [37], has been reported.

Also, insanitary activities such as in-stream flow of domestic wastewater (3KB), poultry wastewaters (2KB, 5KB), slaughterhouse wastewaters, piggery wastewater (2KB, 5KB), livestock manure and litters (throughout the sites except 1TY), wastewater treatment plant effluents (1KT and 5KB), leachates from manhole and community dumpsite (5KT, 5TY), and fertilizers applications (2KT, 5KB, 5TY) offer conditions that could have led to direct input and proliferation of microorganisms, including *P. shigelloides,* along the sampled waters. All sampled sites are livestock watering sites coupled with several other uses with the exception of 1TY (a swimming/recreational hotspot) [51].

The computer-assisted analysis of the ERIC-PCR and (GTG)5-PCR fingerprints of the *P. shigelloides* clustered the isolates into eight and seven clades, respectively, thus suggesting that the *P. shigelloides* from the freshwater resources possessed intra-species or strain diversity. The clustering of strains from different sampling sites together is suggestive of an evolutionary relationship. This is in agreement with [8], who reported that clustered strains originated from different matrices (human and animal sources) or geographical location depicts clonal association. Uniqueness of *P. shigelloides* strains from diarrheic travelers among the Japanese was also demonstrated by Shigematsu et al. [9] using PFGE and DNA macrorestriction digests. Identical DNA-based profile of two pairs of *P. shigelloides* strains from human and animal origin has also been reported [8]. While González-Rey et al. [8] used DNA-based techniques for comparative delineation of a population of single/the same serovar strains, our study did not consider serotypes of the isolates since it has been previously reported that some strains are not serotypeable. Also, subclade (strain clade) diversity was observed in some clades. This explains potential within-clade strain dissimilarity. Notable in this study is the inability of ERIC-PCR to type some strains. Other authors have reported inability of ERIC-PCR to type certain *E. coli* strains [52,53]. In the study of Prabhu et al. [52], 13 out of 40 *E. coli* isolates were not ERIC-PCR typeable, and Ramazanzadeh et al. [54] observed 25 of 230 *E. coli* isolates not typeable by ERIC-PCR. The González-Rey et al. [8] study involved strains that belonged to the same serovar and this might be accounted for the differences observed in our study that involved strains from undifferentiated serovars. (GTG)5-PCR yielded bands for all the strains and thus allowed the differentiation of all *P. shigelloides* strains more efficiently compared with ERIC-PCR.

The relative abundance of the isolates that made up each clade of strain varied significantly. This connotes differences in the occurrence of *P. shigelloides* strains in nature. Heterogeneity in *P. shigelloides* has been reported by many studies, even at the same serovar level [8,9,37]. However, establishing unique identities that discriminate pathogenic strains from nonpathogenic strains has been a challenge. A combination and integrated fingerprinting techniques will be needed to fully identify the intra-species signatures of *P. shigelloides* strains. Besides, strain-specific signatures are now being sought for *P. shigelloides* delineation [54]. Although the ability to cause diarrheal illness has been hypothesized to be universal among *P. shigelloides’* strains from DNA-based fingerprints [9], the resolution of such procedures is not sufficient to support the assumption.

## 5. Conclusions

This study reports intra-species genetic diversity of *P. shigelloides* isolated from freshwaters in the Eastern Cape province, South Africa. The high detection rate of *P. shigelloides* was similar in the three sampled waters (Tyhume, Kat, and Kubusie). The overall *P. shigelloides’* positive isolation rate observed in the study could be attributed to the high level of pollution along the sampled rivers’ courses. Also, abundance and occurrences of *P. shigelloides* strains vary in the aquatic milieu. Both fingerprinting approaches have intra-species/strain characterizing potential for *P. shigelloides’* preliminary grouping purposes. However, ERIC-PCR possessed potentially superior resolution merits over (GTG)5-PCR in *P. shigelloides* intra-species typing.

## Figures and Tables

**Table 1:** Coordinate and name descriptions of the sampling sites.

Sites	Coordinates	Name
1KB	S32°35.429′ E027°21.277′	Stutt. Game Reserve *
1KT	S32°32.891′ E026°45.835′	Seymour
1TY	S32°36.683′; E022°57.612′	Hogsberg
2KB	S32°35.249′ E027°23.916′	StuttTW1 *
2KT	S32°33.870′ E026°43.252′	Katberg
2TY	S32°36.652′ E026°54.564′	Hillfoot *
3KB	S32°35.278′ E027°24.35′	StuttVBridge1 *
3KT	S32°32.450′ E026°40.568′	Balfour
3TY	S32°38.374′ E026°56.163′	Kayalethu
4KB	S32°35.190′ E027°25.415′	StuttEbBridge *
4KT	S32°42.748′ E026°35.682′	Blinkwater
4TY	S32°40.980′ E026°54.080′	Binfield
5KB	S32°35.853′ E027°27.114′	StuttFbridge *
5KT	S32°46.753′ E026°37.250′	Fort Beaufort
5TY	S32°43.223′ E026°51.646′	Melani

* Arbitrary name.

**Table 2:** Molecular confirmation of *P. shigelloides* among randomly selected isolates from IBGA plates.

Sites	Coordinates	Name	Isolates	PCR^+^(%)	PCR^+^/211 × 100
1KB	S32°35.429′ E027°21.277′	Stutt.Game Reserve *	23	07(30.44)	3.32
1KT	S32°32.891′ E026°45.835′	Seymour	89	12(13.5)	5.69
1TY	S32°36.683′; E022°57.612′	Hogsberg	09	08(88.9)	3.79
2KB	S32°35.249′ E027°23.916′	StuttTW1 *	30	06(20.0)	2.84
2KT	S32°33.870′ E026°43.252′	Katberg	65	26(40.0)	12.32
2TY	S32°36.652′ E026°54.564′	Hillfoot *	10	03 (30.0)	1.42
3KB	S32°35.278′ E027°24.35′	StuttVBridge1 *	97	15(15.5)	7.11
3KT	S32°32.450′ E026°40.568′	Balfour	30	20(66.7)	9.48
3TY	S32°38.374′ E026°56.163′	Kayalethu	19	09(47.4)	4.27
4KB	S32°35.190′ E027°25.415′	StuttEbBridge *	42	14(33.33)	6.64
4KT	S32°42.748′ E026°35.682′	Blinkwater	54	13(24.1)	6.16
4TY	S32°40.980′ E026°54.080′	Binfield	59	18(30.5)	8.53
5KB	S32°35.853′ E027°27.114′	StuttFbridge *	23	07(30.4)	3.32
5KT	S32°46.753′ E026°37.250′	Fort Beaufort	119	38(31.9)	18.01
5TY	S32°43.223′ E026°51.646′	Melani	79	19(24.1)	9.01
Total			748	211(28.21)	-

* Arbitrary name.
